# Rivalry between Humans and Coronaviruses: Unanticipated Impact of Omicron

**DOI:** 10.31579/2642-9756/109

**Published:** 2022-03-08

**Authors:** Pascal J. Goldschmidt-Clermont, Alexander J.P. Goldschmidt

**Affiliations:** 1 University of Miami Leonard M. Miller School of Medicine, Dean & Professor Emeritus. Miami, Florida, USA; 2 ALZADY International LLC. Miami, Florida, USA

**Keywords:** rivalry, covid-19, virus, coronavirus, omicron, vaccine, genetic surveillance, mutation, ideation, pandemic, immunity

## Abstract

With our prior Commentary we discussed the rivalry between ideation (humans) and mutations (viruses), (https://www.ncbi.nlm.nih.gov/pmc/articles/PMC8439168/), and more specifically, we described and compared two means of adaptability: collective and focused ideation for humans and self-serving mutation for viruses. The amazingly fast development of new effective and safe vaccines and drugs requires the humankind’s most sophisticated form of ideation ability to respond to threatening stressors such as a dangerous virus like SARS-CoV-2. The essence of what makes us human is that human ideation requires a society of people working towards the same goal and is interdependent on socialization for the sustainability of humankind. In contrast, viruses mutate alone and “selfishly”. The best fit virus for a particular environment, for a particular host, eliminates the competition through successive mutations. The Omicron variant of concern (VoC) is a great example for how higher transmissibility and perhaps, stochasticity, can drive the transmissive success of a virus across an entire host species like humans. With this review, we describe how Omicron has impacted the COVID-19 pandemic in an unanticipated way that could bring an end to it.

## Introduction

Ewen Callaway wrote an article for Nature (November 27, 2021) to report that the World Health Organization (WHO) had designated the strain, known as B.1.1.529, as a variant of concern and named it Omicron [1]. Omicron joined Delta, Alpha, Beta and Gamma on the current WHO list of variants of concern. In particular, the mutation profile of Omicron was of great concern. SARS-CoV-2 is not a very fast mutator, and thus it came as a surprise that its spike protein contained 30 mutations. Suggestions proliferated that Omicron could evade immune responses triggered by vaccines, spread considerably faster than prior variants, and could cause more severe disease than prior variants did. Omicron was first spotted in genome-sequencing surveillance data from Botswana, and rapidly expanded to South Africa, then was spotted also in a rapidly growing series of countries and including the US [1,2].

**Figure 1:**
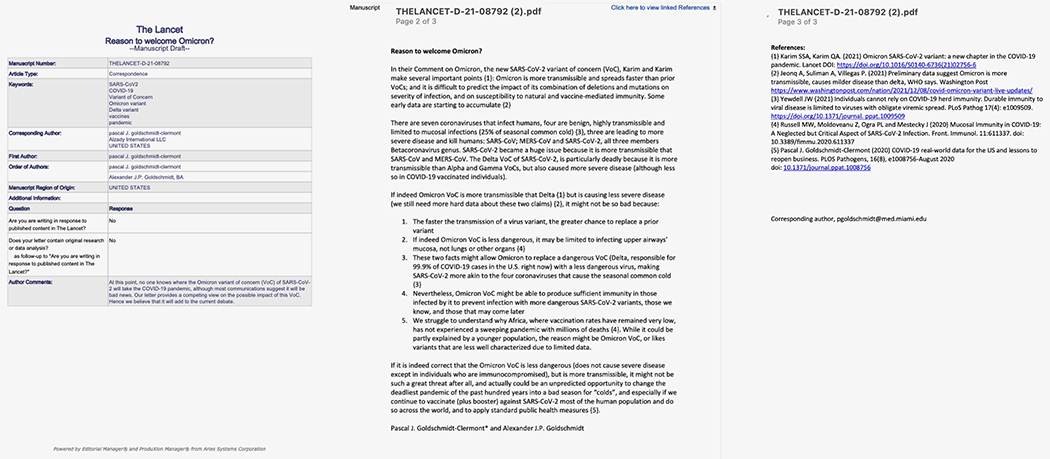
Our letter submitted to the Lancet on December 8, 2021

On December 8, 2021, we submitted a Letter to the Editor of the Lancet [Figure 1]. Karim and Karim had just published an article in the Lancet that made several important points [2]: Omicron is more transmissible and spreads faster than prior VoCs; and it is difficult to predict the impact of its combination of deletions and mutations on severity of infection, and on the effectiveness of natural and vaccine-mediated immunity. Jeonq and colleagues reported in the Washington Post on an early news release from the World Health Organization (WHO) suggesting that that Omicron was indeed more transmissible but could cause milder disease than the Delta VoC [3]. Hence, it was a time of great uncertainty, confusion, and concern that this new VoC could lead to human devastation, health system collapse, and financial freefall for affected nations.

### The Omicron Tsunami

Our Lancet letter was focused on a different perspective, more specifically on a possible silver lining to the Omicron story, it was titled “Reason to welcome Omicron?” [Figure 1]. This is our rationale:

1. There are seven coronaviruses that infect humans, four are highly transmissible and cause collectively 25% of seasonal common colds. And while they may have been dangerous and caused havoc in the past, they are now limited to mucosal infections and thus are benign [4,5].
2. Three coronaviruses are causing severe disease and kill humans mostly because they infect the lungs and can cause acute respiratory distress syndrome (ARDS): SARS-CoV, MERS-CoV and SARS-CoV-2. According to the WHO, 35% of the patients infected with MERS-CoV died [6], and according to the CDC, 9.6% of patients with SARS died [7]. Due to its high transmissibility, while SARS-CoV-2 patients die less frequently (0.6%) than patients with SARS or MERS, the total deaths are substantially greater (921,000) [8]. The Delta VoC of the SARS-CoV-2, was particularly challenging because it is more transmissible than Alpha and Gamma VoCs, but at the same time caused more severe disease especially in younger individuals, although 20-25 times less in COVID-19 fully vaccinated individuals [9].
3. If indeed Omicron VoC is more transmissible that Delta but is causing less severe disease [1,2], it might not be such a disaster because:
  - The faster the transmission of a virus variant, the greater chance to replace a prior variant.
  - If indeed Omicron VoC causes less severe disease, it may be limited to infecting upper airways’ mucosa [5], not lungs (primary cause of death due to COVID-19), or other organs.
4. These two facts allowed Omicron to replace a dangerous VoC (Delta, which was still responsible for 90% of COVID-19 deaths in the U.S. in December 2021) with less dangerous Omicron, making SARS-CoV-2 more akin to the four coronaviruses that cause the seasonal common colds [4].
5. Nevertheless, Omicron VoC might be able to produce sufficient immunity in those infected by it to prevent infection with possibly more dangerous SARS-CoV-2 variants, those we know, and those that may come later [10,11]
6. Finally, we have struggled to understand why Africa, where vaccination rates have remained very low, has not experienced a sweeping pandemic with millions of deaths as had been predicted by some experts [12]. While it could be partly explained by a younger population [13], the reason might be infection with Omicron VoC, or several prior consecutive variants that have been missed by genomic surveillance due to limited data, which could also explain the rather large number of new mutations on Omicron.

## Discussion

We had anticipated that the Omicron VoC, being less dangerous (rarely causes severe disease except for victims that are immunocompromised, susceptible and elderly) , but more transmissible, would actually replace the much more dangerous Delta VoC (Figure 1). Our expectation was that this pandemic would end after a succession of battles between slightly improved mutated variants and increasingly accurate and precise vaccines. However, the unanticipated tsunami of Omicron infections created an unprecedented opportunity to possibly end the deadliest pandemic of the past hundred years. Lancet decided not to publish our “Letter” eight weeks after submission, a slow mail system indeed.

One of the greatest concerns with omicron was the large number of mutations [1,2], some similar to prior variants (including Delta VoC), some we had never seen before. Another concern with Omicron, most likely resulting from the many mutations, is its ability to evade vaccine and prior infection immunity. Full vaccination confers at best 10-20% effectiveness in preventing infections with Omicron, and adding a booster might improve effectiveness some. The rate of reinfection with Omicron for those with prior infections with other SARS-CoV-2 variants is increased by nearly ten folds. It is possible that Omicron has evolved for a while in Africa where it remained undetected while infecting the mostly unvaccinated population and protecting it from more dangerous variants. The BA.1 variant that was discovered in Botswana could be a more evolved variant of the B.1.1.529 Pango lineage. Genomic surveillance has identified a BA.2 variant, and there were probably many Omicron variants before B.1.1.529, BA.1 and BA.2 as suggested by the latest BA. 3 discovery in northwestern South Africa [14].

## Conclusion

Due to Omicron’s high transmissibility and its low morbidity and mortality, it is a less dangerous variant that has spread massively across the world. The strong elicited immune response post Omicron infection along with COVID-19 vaccines and boosters, while continuing to apply standard public health measures [15], may keep COVID-19 infections at bay, and for the foreseeable future.

## Figures and Tables

**Figure 2:**
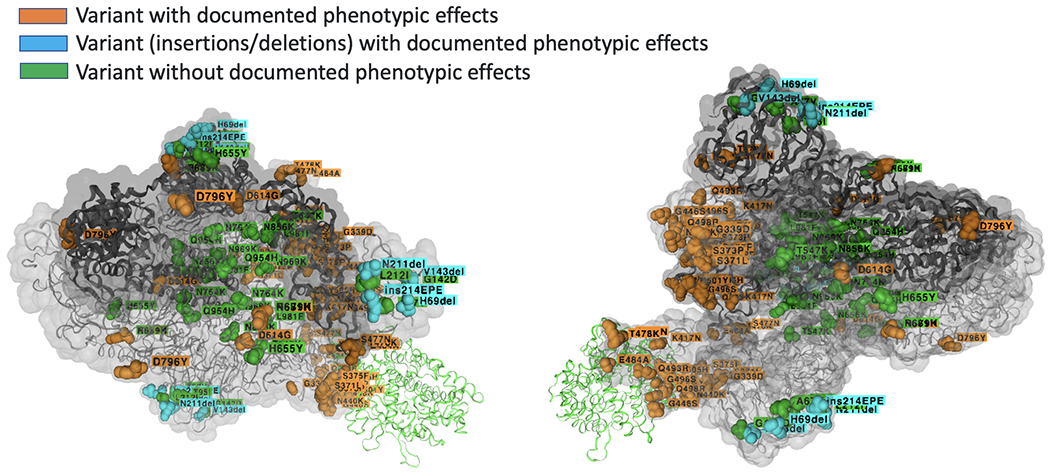
GISAID three-dimensional (3-D) structure of the spike glycoprotein with Amino Acid Changes [16]. SARS-CoV-2 spike glycoprotein trimer (3 units shown, gray ribbons) is shown in complex with its human host cell receptor ACE2 (1 unit shown, light green ribbon). Amino Acid positions with changes in the B.1.1.529+BA (Omicron) lineages are indicated as colored balls. Changes with documented phenotypic effects according to the literature are colored in light orange or blue for insertions and deletions while other changes without documented phenotypic effects are colored in green.
